# Perception of worry of harm from air pollution: results from the Health Information National Trends Survey (HINTS)

**DOI:** 10.1186/s12889-022-13450-z

**Published:** 2022-06-25

**Authors:** Samantha Ammons, Hayley Aja, Armen A. Ghazarian, Gabriel Y. Lai, Gary L. Ellison

**Affiliations:** 1 Epidemiology and Genomics Research Program, Division of Cancer Control and Population Sciences, Department of Health and Human Services, National Cancer Institute, National Institutes of Health, MD Bethesda, USA; 2 Extramural Research Branch, Extramural Research and Partnerships Division, Office of Science Advisor, Policy, and Engagement, U.S. Environmental Protection Agency, Washington, DC, USA

**Keywords:** Air pollution, Health communication, Cancer, HINTS

## Abstract

**Background:**

Air pollution exposure has been associated with a multitude of diseases and poses a significant concern to public health. For targeted environmental risk communication and interventions to be effective, it is important to correctly identify characteristics associated with worry of harm from air pollution.

**Methods:**

Using responses from 3,630 participants of the Health Information National Trends Survey 4 Cycle 2, we assessed worry of harm from exposure to indoor (IAP) and outdoor (OAP) air pollution separately. Multinomial logistic regression models were used to calculate odds ratios and 95% confidence intervals.

**Results:**

Hispanics were more likely to worry about harm from IAP and OAP compared to non-Hispanic whites. Participants who lived in metropolitan counties were more likely to worry about harm from IAP and OAP compared to those who lived in rural counties. Finally, those who believed their chance of getting cancer was high were more likely to worry about harm from IAP and OAP compared to those who thought their likelihood of getting cancer was low.

**Conclusions:**

Worry of harm from IAP and OAP varied across sociodemographic and cancer-related characteristics. Public health professionals should consider these characteristics when developing targeted environmental risk communication and interventions.

**Supplementary Information:**

The online version contains supplementary material available at 10.1186/s12889-022-13450-z.

## Background

Air pollution refers to both man-made and naturally occurring pollutants in the air we breathe. Although air pollution is often invisible and odorless, exposure to it is ubiquitous and a threat to public health. Air pollutants include, but are not limited to noxious gases, fine particles produced by the burning of fossil fuels, and tobacco smoke. Air pollution increases the risk for developing heart and respiratory diseases [17]. In addition, air pollution exposure has been associated with the risk for developing some cancers, the most common being lung [18, 24, 54], breast [48, 50], and bladder [42]. A meta-analysis of 30 cohort studies from 14 countries found that across numerous cancers, exposure to particulate matter (PM) 2.5, PM 10, and nitrogen dioxide was associated with an increase in cancer mortality [26].

There have been efforts at the policy level to control indoor and outdoor air pollution. Some examples in recent years include the Clean Air Act of 1970 in the United States (U.S.) and World Health Organization interim targets concerning PM control on cardiovascular disease mortality, both of which have led to a decrease in particle pollution and ground-level ozone pollution [33, 47]. Nevertheless, approximately seven million people die from air pollution exposure around the world every year [23]. Further, adverse health effects due to air pollution exposure are often concentrated in among those who have been characterized as having low socioeconomic status and individuals suffering from pre-existing conditions [38].

It is important to distinguish between outdoor and indoor air pollution as they each pose different health risks. Outdoor air pollution refers to pollutants such as ground-level ozone, noxious gases, and fine particles produced by burning fossil fuels by motor vehicles and power plants. Exposure to chemicals found in air pollution are harmful to humans because these chemicals are often small enough to penetrate deep inside the lungs and cause a variety of adverse reactions such as early impairment of airway function, chronic system inflammation, oxidative stress, and lung cancer [4, 18, 52].

Indoor air pollution refers to air pollutants found inside our homes, schools, and other building environments. Sources of the most common indoor air pollutants from economically developing countries often differ from the sources found in economically developed countries like the US. Common indoor air pollutants in the US are derived from secondhand smoke, carbon monoxide exposure from gas and wood stoves primarily used in rural areas [51], and radon exposure from building foundations [49]. Additionally, volatile organic compounds (VOCs) are chemicals found in many household products such as aerosol sprays, cleaners, and pesticides contain toxic and carcinogenic compounds such as benzene and toluene in the air and water [13]. Overall, indoor air pollution remains a major public health concern because people often spend most of their time indoors at work or inside their homes, further intensifying the concentration of exposure to harmful indoor pollutants. In the United States, specifically, “radon, a major indoor air pollutant, is the second leading cause of cancer and responsible for 15,000 to 20,000 deaths each year” [37].

Effective risk communication and targeted interventions, such as household and behavior change interventions, can be productive tools to mitigate the adverse effects of air pollution exposure. Additionally, it is important to inform the public about the risks associated with air pollution in ways that do not create a sense of unwarranted indifference, while at the same time, not creating unwarranted anxiety [19]. Nevertheless, due to individual variability in air pollution exposure and public health risk across the US, developing a one-size-fits-all risk communication strategy along with targeted interventions can be challenging and ineffective. In order for risk communication to be effective, information should be current and appropriate to the targeted audience [46].

Several studies have examined participants’ perception of harm due to air pollution exposure [16, 32, 35]. However, to our knowledge, there have been no studies that have explicitly measured one’s worry of harm from indoor and outdoor air pollution exposure. Additionally, studies regarding air pollution perception have not been conducted in the U.S. nor have they explored cancer-related characteristics, such as family cancer history and perception of one’s cancer risk [3, 29, 40]. Lastly, many of these studies were focused on either indoor or outdoor air pollution separately or used the term “air pollution” without distinguishing whether it was indoor or outdoor [21, 27, 41]. The purpose of this study was to examine sociodemographic and cancer-related characteristics associated with worry of harm from exposure to indoor air pollution (IAP) and outdoor air pollution (OAP) using data from a nationally representative survey in the US.

## Methods

The Health Information National Trends Survey (HINTS) is a nationally representative survey supported by the National Cancer Institute that aims to describe cancer-related knowledge, beliefs, attitudes, and behaviors of adults in the US. HINTS uses a probability sample of U.S. telephone numbers to reach a cross-section of the adult, non-institutionalized, and civilian U.S. population. Data were collected using a computer-assisted telephone interview system administered in English or Spanish. Response rates were calculated using the Response Rate 2 (RR2) formula of the American Association of Public Opinion Research [36]. We utilized data from the 2012 iteration of the HINTS 4 Cycle 2. There was a total of 3,630 participants in the 2012 iteration of the HINTS with an overall response rate of 40%. Racial and ethnic minorities were oversampled to increase the precision of estimates for minority sub-populations. Additional details on the HINTS methodology as well as access to the complete HINTS datasets can be found on hints.cancer.gov as well as previous literature and reports [1, 56].

For this study, we used the questions “How much do you worry that indoor air pollution will harm your health?” and “How much do you worry that outdoor air pollution will harm your health?”. Participants were able to respond: “not at all”, “a little”, “somewhat”, or “a lot” to each question. Due to the lack in significant differences in ORs for the “a little” and “somewhat” responses for indoor and outdoor air pollution and the variables of interest, we combined the two responses into “some or a little” to create a total of three response categories: “a lot”, “some or a little”, and “not at all” (data not shown).

Sociodemographic variables included sex (male and female), age (18–34, 35–49, 50–64, and ≥ 65), race/ethnicity (Hispanic, non-Hispanic white, non-Hispanic black, and Asian, Pacific Islander, other), whether someone was born in the United States, education level (high school diploma or less, some college or post-high school vocational training, bachelor’s degree or more), annual household income (≤ $20,000, $20,000–34,999, $35,000–49,000, $50,000–74,000, and ≥ $75,000), smoking status (never smoker, former smoker, and current smoker), and whether respondents resided in a rural or metropolitan county. For our analysis, non-metropolitan and metropolitan residence was determined based on Rural Urban Continuum Codes in which categories 1 through 3 (counties with 250,000 residents or more) were considered “metropolitan” and categories 4 through 9 (counties with less than 250,000 residents) were considered “non-metropolitan” [57]. In addition to sociodemographic variables, we also examined cancer-related variables including family history of cancer (No, Not Sure, Yes), previous diagnosis of lung disease, which included chronic lung disease, asthma, emphysema, and/or chronic bronchitis (No, Yes), and perceived likelihood of getting cancer (Very Unlikely or Unlikely, Neither Likely or Likely, and Very Likely or Likely).

Bivariate analyses of sociodemographic variables and cancer-related characteristic variables by worry of harm from IAP and OAP were conducted using the Wald chi-squared test and unadjusted odds ratios and 95% confidence intervals were calculated for each of the variables. In addition, a sensitivity analysis was conducted to confirm there were no major differences in estimates of odds ratios between the separate “somewhat” and “a little” response categories and the combined “some or a little” response category we created for IAP and OAP when compared to the “not at all” response category. There were no major differences in odds ratios between the original IAP and OAP discrete “somewhat” and “a little” response categories and the combined category, therefore we used the combined response (“some or a little”) category for IAP and OAP for our analysis. The final response categories were: “not at all”, “some or a little”, and “a lot” for IAP and OAP. A *p*-value of less than or equal to 0.05 was used to determine statistical significance. Multinomial logistic regression was used to calculate odds ratios (OR) and 95% confidence intervals (CI) for the association between sociodemographic and cancer-related variables and worry of harm from IAP and OAP (separately). Participants with any missing values were removed from the logistic regression analysis. All models were adjusted for demographic variables including sex, race/ethnicity, education, and non-metropolitan-metropolitan residence. SAS (version 9.4; Cary, NC) was used to conduct all statistical analyses.

## Results

The distribution of demographic factors for the sample population and the weighted population are presented in Table 1. Among the sample population, a little more than half consisted of women (*n* = 2,172, 51.4%). Nearly two-thirds (62.7%) of the respondents were non-Hispanic white, while 1,057 (37.6%) had completed some college or post-high school vocational training, and 926 (31.0%) had an annual household income greater than $75,000. Most respondents (68.5%) reported a known family history of cancer while 20% of respondents (*n* = 621) believed they had a very likely or likely chance of getting cancer themselves. The prevalence of respondents who had at least some or a little worry of harm for IAP and OAP was 55.2% and 57.0%, respectively. Additionally, the prevalence of respondents who worried “a lot” about harm from IAP and OAP were 12.7% and 17.3%, respectively.

**Table 1:** Sociodemographic and Cancer-Related Variable Summary

CHARACTERISTIC	N^a^	%^b^
	(*N* = 3630)	
Sex		
Male	1390	48.6
Female	2172	51.4
Age		
18–34	529	30.5
35–49	845	26.4
50–64	1168	25.6
65 +	970	17.4
Race/Ethnicity		
Non-Hispanic White	2043	62.7
Hispanic	511	15.7
Non-Hispanic Black	496	15.2
Asian, Pacific Islander, and Other	208	6.4
Born in the USA		
Yes	3056	86.6
No	513	14.4
Education		
High School Diploma or Less	1104	33.8
Some College or Post-High School Vocational Training	1057	37.6
College Graduate or Post-Graduate Degree	1380	28.6
Annual Household Income		
Less than $20,000	740	21.9
$20,000 – 34,999	501	14.9
$35,000 – 49,999	459	15.5
$50,000—$74,999	524	16.8
More than $75,000	926	31.0
Smoking Status		
Never Smoker	2052	58.6
Former Smoker	939	22.7
Current Smoker	586	18.7
Reside in Metropolitan or Non-Metropolitan County		
Non-Metropolitan County	543	16.3
Metropolitan County	3087	83.7
Previous Family Cancer History		
No/Don’t Know	870	28.0
Yes	2412	72.0
Previous Lung Disease Diagnosis^c^		
No	2994	84.6
Yes	500	15.4
Likelihood of Getting Cancer		
Very Unlikely or Unlikely	992	35.5
Neither Unlikely nor Likely	1371	44.4
Very Likely or Likely	621	20.0

^a^N is based on the HINTS respondents; may not sum to total due to missing values

^b^% is based on the estimated percentage of the U.S. adult population

^c^This includes chronic lung disease, asthma, emphysema, and/or chronic bronchitis

### Bivariate analyses

Bivariate associations between worry of harm from IAP and OAP with sociodemographic and cancer characteristic variables are presented in Tables 2 and 3, respectively. Worry of harm from IAP was statistically significantly associated with race/ethnicity, education, non-metropolitan-metropolitan residence, and likelihood of getting cancer (Table 2). Worry of harm from OAP was statistically significantly associated with sex, race/ethnicity, whether someone was born in the U.S., education, smoking status, non-metropolitan-metropolitan residence, previous family history of cancer, previous lung disease diagnosis, and likelihood of getting cancer (Table 3). The magnitude of association was presented as unadjusted odds ratios and 95% confidence intervals calculated for worry of harm from IAP and OAP with sociodemographic and cancer characteristic variables (see: Supplemental Table).

**Table 2:** Bivariate analysis of select characteristics and worry of harm from IAP

**Total**	Total	A lot	Some or A Little	Not at all	
	N^a^ (%)^b^	N (%)	N (%)	N (%)	χ2-test
	3486 (100.0)	441 (12.7)	1924 (55.2)	1121 (32.2)	*P*-value
**Sex**					
Male	1344 (48.8)	147 (42.4)	726 (48.0)	471 (52.6)	0.09
Female	2088 (51.2)	286 (57.6)	1168 (52.0)	634 (47.4)	
**Age**					
18–34	525 (31.1)	55 (31.9)	275 (29.7)	195 (33.3)	0.26
35–49	834 (26.8)	118 (29.4)	477 (27.8)	239 (23.9)	
50–64	1141 (25.8)	147 (24.8)	646 (27.2)	348 (23.6)	
65 +	901 (16.4)	107 (14.0)	479 (15.3)	315 (19.1)	
**Race/Ethnicity**					
Non-Hispanic White	2009 (67.6)	164 (50.3)	1131 (68.0)	714 (73.6)	< 0.0001
Hispanic	481 (14.2)	111 (23.8)	244 (13.7)	126 (11.4)	
Non-Hispanic Black	484 (11.0)	75 (13.0)	257 (10.6)	152 (10.9)	
Asian, Pacific Islander, and Other	204 (7.2)	32 (12.9)	128 (7.7)	44 (4.0)	
**Born in the USA**					
Yes	2971 (85.5)	295 (65.1)	1658 (85.9)	1018 (92.9)	< 0.0001
No	476 (14.5)	137 (34.9)	245 (14.1)	94 (7.1)	
**Education**					
High School Diploma or Less	1021 (32.7)	154 (42.6)	542 (31.8)	325 (30.4)	0.03
Some College or Post-High School Vocational Training	1039 (38.1)	148 (36.2)	582 (39.5)	309 (36.4)	
College Graduate or Post-Graduate Degree	1364 (29.2)	124 (21.3)	768 (28.7)	472 (33.1)	
**Annual Household Income**					
Less than $20,000	694 (21.5)	131 (26.5)	363 (21.8)	200 (19.1)	0.39
$20,000 – 34,999	489 (14.7)	71 (16.5)	283 (15.1)	135 (13.1)	
$35,000 – 49,999	451 (15.4)	51 (14.3)	259 (16.6)	141 (13.7)	
$50,000—$74,999	518 (17.1)	51 (15.4)	291 (15.6)	176 (20.3)	
More than $75,000	918 (31.4)	82 (27.3)	500 (30.9)	336 (33.9)	
**Smoking Status**					
Never Smoker	1981 (58.8)	267 (65.9)	1109 (59.4)	605 (54.9)	0.20
Former Smoker	907 (22.8)	100 (18.5)	494 (22.7)	313 (24.6)	
Current Smoker	570 (18.4)	71 (15.6)	310 (17.8)	189 (20.5)	
**Non-Metropolitan-Metropolitan Residence**					
Non-Metropolitan	522 (16.5)	51 (14.6)	288 (14.4)	183 (21.1)	< 0.01
Metropolitan	2964 (83.5)	390 (85.4)	1636 (85.6)	938 (78.9)	
**Previous Family Cancer History**					
No	835 (26.0)	103 (30.9)	469 (25.4)	263 (25.2)	0.52
Not Sure	231 (7.5)	33 (6.4)	136 (7.1)	62 (8.6)	
Yes	2339 (66.5)	290 (62.7)	1270 (67.5)	779 (66.2)	
**Previous lung disease diagnosis**^c^					
Not Diagnosed with Lung Disease	2897 (84.7)	348 (79.6)	1583 (84.2)	966 (87.6)	0.14
Diagnosed with Lung Disease	487 (15.3)	73 (20.4)	290 (15.8)	124 (12.4)	
**Likelihood of Getting Cancer**					
Very Unlikely or Unlikely	964 (35.6)	126 (42.1)	545 (36.5)	293 (31.5)	< 0.001
Neither Unlikely nor Likely	1348 (45.2)	132 (33.8)	782 (48.0)	434 (44.3)	
Very Likely or Likely	589 (19.3)	84 (24.0)	297 (15.6)	208 (24.2)	

^a^N is based on the HINTS survey respondents who answered the IAP question in HINTS survey; may not sum to total due to missing values

^b^% is based on the estimated % of U.S. adult population

^c^This includes chronic lung disease, asthma, emphysema, and/or chronic bronchitis

**Table 3:** Bivariate analysis of select characteristics and worry of harm from OAP

**Total**	Total	A lot	Some or A Little	Not at all	
	N^a^ (%)^b^	N (%)	N (%)	N (%)	χ2-test
	3511 (100.0)	608 (17.3)	1999 (57.0)	904 (27.8)	*P*-value
**Sex**					
Male	1355 (48.7)	201 (40.6)	758 (47.6)	396 (55.6)	< 0.01
Female	2101 (51.3)	393 (59.4)	1216 (52.4)	492 (44.4)	
**Age**					
18–34	526 (31.0)	65 (25.6)	320 (31.4)	141 (33.2)	0.40
35–49	836 (26.8)	151 (30.1)	491 (27.1)	194 (24.3)	
50–64	1144 (25.7)	206 (28.1)	648 (25.8)	290 (24.0)	
65 +	914 (16.5)	165 (16.2)	494 (15.6)	255 (18.5)	
**Race/Ethnicity**					
Non-Hispanic White	2012 (67.6)	234 (47.9)	1202 (69.5)	576 (74.5)	< 0.0001
Hispanic	486 (14.3)	135 (22.0)	257 (14.6)	94 (9.4)	
Non-Hispanic Black	489 (11.0)	110 (13.7)	256 (9.8)	123 (12.1)	
Asian, Pacific Islander, and Other	202 (7.1)	53 (16.4)	113 (6.1)	36 (4.0)	
**Born in the USA**					
Yes	2988 (85.5)	428 (66.0)	1735 (87.0)	825 (93.4)	< 0.0001
No	482 (14.5)	171 (34.0)	243 (13.0)	68 (6.6)	
**Education**					
High School Diploma or Less	1041 (33.0)	207 (44.6)	535 (28.2)	299 (36.3)	< 0.001
Some College or Post-High School Vocational Training	1040 (38.0)	181 (31.5)	604 (40.7)	255 (35.9)	
College Graduate or Post-Graduate Degree	1366 (29.0)	203 (23.8)	828 (31.1)	335 (27.8)	
**Annual Household Income**					
Less than $20,000	704 (21.7)	165 (27.7)	369 (19.9)	170 (22.1)	0.21
$20,000 – 34,999	492 (14.7)	91 (19.4)	289 (13.4)	112 (14.7)	
$35,000 – 49,999	453 (15.4)	78 (15.0)	259 (16.4)	116 (13.4)	
$50,000—$74,999	518 (16.9)	62 (12.2)	316 (17.2)	140 (18.9)	
More than $75,000	917 (31.3)	125 (25.8)	546 (33.0)	246 (30.9)	
**Smoking Status**					
Never Smoker	1995 (58.6)	371 (66.0)	1147 (59.5)	477 (52.5)	< 0.01
Former Smoker	915 (22.8)	144 (19.9)	532 (23.9)	239 (22.4)	
Current Smoker	573 (18.5)	89 (14.1)	309 (16.6)	175 (25.1)	
**Non-Metropolitan-Metropolitan Residence**					
Non-Metropolitan	520 (16.4)	64 (12.4)	287 (14.8)	169 (21.9)	< 0.01
Metropolitan	2991 (83.6)	544 (87.6)	1712 (85.2)	735 (78.1)	
**Previous Family Cancer History**					
No	845 (26.1)	149 (29.5)	465 (23.1)	231 (30.2)	0.02
Not Sure	231 (7.4)	48 (6.7)	131 (6.7)	52 (9.3)	
Yes	2349 (66.5)	392 (63.7)	1356 (70.2)	601 (60.5)	
**Previous lung disease diagnosis**^c^					
Not Diagnosed with Lung Disease	2915 (84.5)	462 (75.7)	1675 (86.2)	778 (86.0)	< 0.01
Diagnosed with Lung Disease	491 (15.5)	122 (24.3)	271 (13.8)	98 (14.0)	
**Likelihood of Getting Cancer**					
Very Unlikely or Unlikely	973 (35.8)	186 (43.8)	573 (37.4)	214 (27.9)	< 0.001
Neither Unlikely nor Likely	1350 (44.8)	185 (33.6)	802 (45.5)	363 (47.6)	
Very Likely or Likely	593 (19.4)	121 (22.6)	295 (16.1)	177 (24.6)	

^a^N is based on the HINTS survey respondents who answered the OAP question in HINTS survey, may not sum to total due to missing values

^b^% is based on the estimated % of U.S. adult population

^c^This includes chronic lung disease, asthma, emphysema, and/or chronic bronchitis

### Multivariable analyses

The association of worry of harm from IAP and OAP with select sociodemographic and cancer characteristics is presented in Table 4. Asians, Pacific Islanders, and those from other races (OR: 4.74, 95% CI: 1.93 – 11.67) and Hispanics (OR: 2.44, 95% CI: 1.34 – 4.43) were more likely to worry “a lot” about harm from IAP compared to non-Hispanic whites. Those who obtained a high school diploma or less were more likely to worry “a lot” about harm from IAP compared to those who were a college graduate or obtained a post-graduate degree (OR: 2.38, 95% CI: 1.39 – 4.08). Additionally, those who believed they were very likely or likely to get cancer in their lifetime were approximately two times more likely to worry “a lot” about harm from IAP compared to those who believed they were neither unlikely or likely to get cancer in their lifetime (OR: 1.99, 95% CI: 1.20 – 3.29). Lastly, respondents who were born outside the U.S. were more likely to worry “some or a little” (OR: 1.90, 95% CI: 1.11 – 2.93) and “a lot” (OR: 5.64, 95% CI: 3.52 – 9.01) about IAP than respondents who were born in the U.S.

**Table 4:** Multinomial Logistic Regression Analysis of IAP and OAP

**Characteristic**	**Indoor Air Pollution**				**Outdoor Air Pollution**			
	**Some or a little vs Not at all**		**A lot vs Not at all**		**Some or a little vs Not at all**		**A lot vs Not at all**	
	**OR**	**95% CI**	**OR**	**95% CI**	**OR**	**95% CI**	**OR**	**95% CI**
**Sex**								
Male	1.00	-	1.00	-	1.00	-	1.00	-
Female	1.18	0.92—1.50	1.54	1.00—2.36	1.37	0.98—1.91	1.98	1.25—3.15
**Race/Ethnicity**								
Non-Hispanic White	1.00	-	1.00	-	1.00	-	1.00	-
Hispanic	1.18	0.77—1.79	2.50	1.28—4.88	1.65	1.03—2.66	3.40	1.79—6.47
Non-Hispanic Black	0.98	0.70—1.37	1.53	0.92—2.56	0.83	0.51—1.35	1.56	0.89—2.75
Asian, Pacific Islander, and Other	2.31	1.23—4.32	5.24	2.06—13.35	1.77	0.88—3.59	7.63	3.34—17.45
**Born in the USA**								
Yes	1.00	-	1.00	-	1.00	-	1.00	-
No	1.82	1.10—2.99	5.61	3.39—9.28	1.65	0.93—2.93	4.59	2.66—7.92
**Education**								
College Graduate or Post- Graduate Degree	1.00	-	1.00	-	1.00	-	1.00	-
High School Diploma or Less	1.39	1.03—1.88	2.46	1.40—4.31	0.77	0.54—1.10	1.63	0.97—2.74
Some College or Post-High School Vocational Training	1.35	0.99—1.84	1.59	0.96—2.64	1.03	0.76—1.41	1.07	0.69—1.65
**Non-Metropolitan-Metropolitan Residence**								
Non-Metropolitan	1.00	-	1.00	-	1.00	-	1.00	-
Metropolitan	1.66	1.26—2.19	1.69	0.87—3.30	1.44	1.02—2.04	1.73	1.00—2.98
**Previous Family Cancer History**								
No	1.00	-	1.00	-	1.00	-	1.00	-
Not Sure	0.88	0.48—1.60	0.56	0.23—1.34	1.12	0.60—2.08	0.83	0.35—1.96
Yes	1.04	0.76—1.41	0.85	0.52—1.38	1.64	1.20—2.26	1.24	0.76—2.00
**Previous lung disease diagnosis**^b^								
Not Diagnosed with Lung Disease	1.00	-	1.00	-	1.00	-	1.00	-
Diagnosed with Lung Disease	1.27	0.80—2.03	1.98	1.00—3.94	0.96	0.58—1.57	2.16	1.20—3.86
**Likelihood of Getting Cancer**								
Neither Unlikely nor Likely	1.00	-	1.00	-	1.00	-	1.00	-
Very Unlikely or Unlikely	0.56	0.42—0.75	1.01	0.55—1.88	0.71	0.49—1.04	1.03	0.60—1.78
Very Likely or Likely	1.13	0.81—1.57	1.93	1.15—3.23	1.51	1.10—2.08	2.62	1.57—4.38

^a^Adjusted for Race, Education, Sex, and Non-Metropolitan-Metropolitan Residence

^b^This includes chronic lung disease, asthma, emphysema, and/or chronic bronchitis

Women were more likely to worry “a lot” about harm from OAP compared to men (OR: 1.95, CI: 1.24 – 3.08). Asians, Pacific Islanders, and those from other races (OR: 6.25, 95% CI: 2.92 – 13.36) and Hispanics (OR: 3.02, 95% CI: 1.78 – 5.15) were more likely to worry “a lot” about harm from OAP compared to non-Hispanic whites. Those who obtained a high school diploma or less were more likely to worry “a lot” about harm from OAP compared to those who obtained a college graduate or post-graduate degree (OR: 1.64, 95% CI: 1.03 – 2.61). Participants who lived in an metropolitan county were more likely to worry “a lot” about harm from OAP compared to those who live in non-metropolitan counties (OR: 1.88, 95%CI: 1.08 – 3.28). Respondents who were born outside the U.S. were approximately 4.60 times more likely than those born in the U.S. to worry about harm from OAP (OR: 4.60, 95%CI: 2.78 – 7.63). Additionally, those who had a diagnosis of lung disease were more likely to worry “a lot” about harm from OAP compared to those who did not have a diagnosis of lung disease (OR: 2.10, 95% CI: 1.20 – 3.68). Finally, those who believed they were very likely or likely to get cancer in their lifetime were approximately 2.5 times more likely to worry “a lot” about harm from OAP compared to those who believed they were neither unlikely or likely to get cancer in their lifetime (OR: 2.56, 95% CI: 1.57 – 4.19).

## Discussion

Data from the 2012 HINTS suggest that there are several subgroups of the U.S. population that worried more about harm from IAP and OAP compared to others. The subgroups included women, Hispanics, Asians/Pacific Islanders, and other races (in comparison to non-Hispanic Whites), people born outside the United States, those who lived in metropolitan counties, as well as people who have a previous family cancer history and/or lung disease diagnosis (in comparison to those who do not have a previous family cancer history and/or lung disease diagnosis). Our findings indicate that such groups should be considered when developing air pollution risk communication and intervention strategies. Additionally, our results suggest that more communication regarding the prevalence and risks of indoor air pollution exposure is needed.

Our finding that women were nearly twice as likely as men to be worried “a lot” about outdoor air pollution is consistent with what the literature suggests about environmental risks and women. Other studies have found that women, especially those with children, are more aware of and concerned about environmental risks [14, 27]. One possible reason for this is because air pollution has been associated with poor reproductive health outcomes including infertility, miscarriage, and stillbirth [11, 20]. Moreover, one case control study found that prenatal exposure to ambient PM_2,5_ increased mental stress for pregnant women [28].

Another noteworthy finding in this study was that respondents born outside the U.S. worried a lot about harm from IAP and OAP compared to those born in the U.S. According to 2012 immigration data, the year the HINTS respondents took this survey, more than half of U.S. foreign-born residents came from either Mexico (28.2%) or East and South Asia (25.6%), regions with high rates of air pollution exposure [7, 43]. Further, the Health Effects Institute 2020 State of Global Air Report found that East and South Asia ranked among the highest for household indoor air pollution as well as ambient air pollution exposure in the world [23]. Additionally, the vast majority of literature about the perception of air pollution was based in Low and Low Middle Income Countries, namely China [22, 30, 32, 58] and Mexico [2, 3, 6]. This finding has been consistent for the past several decades [9]. Nevertheless, it is plausible that residence in a country with high pollution could lead one to worry less about air pollution in their host country after migration; however, the literature on this topic is quite limited and we found no evidence of this relationship. Furthermore, perceptions of worry being influenced by thoughts of pollution in one’s home country may explain why those not born in the U.S. worried more about harm from air pollution exposure in comparison to those born in the U.S. However, more work is needed to understand this phenomenon.

Worry of harm from air pollution exposure also varied by geographic area of residence in this study. Although we found no significant differences with region (data not shown), across IAP and OAP, participants who resided in metropolitan counties were more concerned about air pollution exposure than those who resided in non-metropolitan counties. This finding is consistent with air pollution literature concerning metropolitan areas which suggests that residents in metropolitan areas have significantly more air pollution exposure compared to non-metropolitan residents [8, 34, 39, 55]. Ozone is a significant source of air pollution in non-metropolitan areas and most of the worry of harm we observed was in metropolitan areas [31]. This underscores the need for education around sources of air pollution in metropolitan and non-metropolitan areas.

We also observed that people with cancer-related characteristics, such as a family history of cancer, lung disease diagnosis, and having beliefs they will get cancer is another subgroup of participants that worried about harm from air pollution exposure. This finding is consistent with the literature that indicates an association of cancer and lung disease incidence with air pollution exposure [18, 44], as well as cancer survivorship and air pollution exposure [15, 52]. Additionally, 82.24% of respondents with family history of cancer believe they were very likely or likely to get cancer (data not shown), further suggesting that people with cancer-related characteristics see themselves at risk for adverse health outcomes related to air pollution exposure.

The association between some sociodemographic and cancer-related characteristics and air pollution differed for IAP and OAP. Characteristics of respondents that were statistically significant for OAP and not IAP were sex, previous family history of cancer, and having a previous diagnosis of lung disease. A lack of understanding of the nuances that distinguish indoor from outdoor air pollution could potentially explain these observed differences. Much of the public discourse on air pollution focuses on indoor and outdoor air pollution as one entity or only focuses on outdoor air pollution. Consequently, research and subsequent policy interventions focused on the health impacts of indoor air pollution lags behind that of outdoor air pollution [5]. This is an important finding because indoor pollution on average poses considerable serious health risks such as lung cancer, asthma, and heart disease [25, 37]. It is important that air pollution risk communication effectively distinguishes differences between indoor and outdoor air pollution, particularly the sources and health risks associated with each category.

## Conclusions

### Strengths and limitations

To our knowledge, this is the first study to evaluate the association between worry of harm from indoor and outdoor air pollution and sociodemographic and cancer-related variables. Additionally, the environmental questions we used from the 2012 iteration of HINTS have not been previously published. One strength of this study is that HINTS relies on multiple methods to reduce non-response including an initial mailing of the questionnaire, a reminder card, and up to three additional questionnaire mailings. Other strengths include our ability to account for a wide range of related covariates, including important risk factors and potential confounders such as geographic area of residence, and education. Nonetheless, there were some limitations to this study, including the cross-sectional study design. Cross-sectional studies limit the ability to evaluate causality and to compare responders with non-responders [53]. Additionally, these data were collected in 2012, and the respondents’ perception may have changed with time. Another potential limitation is that HINTS did not include an explicit definition of indoor and outdoor air pollution. Participants may not fully understand the inherent differences (in causes and health risks) of both air pollution types. Moreover, single items to measure such indoor and outdoor pollution may have lowered reliability and diminished the chances of identifying significant relationships [12].

### Implications and future research

Risk communication such as the Air Quality Index (AQI) often does not consider sociodemographic and cancer-related characteristics when developing messaging and interventions and primarily or solely focus on concentration of pollutants. The sociodemographic and cancer-related characteristics identified in this study can be applied to future research with more recent data. Such research can be used to inform environmental agencies as well as local, state, and federal governments regarding targeted communication and encourage various subgroups to take protective actions against air pollution exposure including staying indoors during poor outdoor air quality days and changing air filters regularly to limit indoor air pollution exposure [45]. Risk communication should also consider perception of air pollution exposure within microenvironments including one’s commute. For example, research suggesting that changing one’s bicycle route to areas with less motorized traffic may decrease exposure to particulate matter, odor, and nasopharyngeal irritation [10] could be included in communication materials as a protective action against air pollution exposure. It is important to understand what factors influence adherence to communication tools such as public service announcements, the AQI, etc. Future research is warranted which includes greater study participation, more detailed questions about indoor and/or outdoor air pollution knowledge, longitudinal data collection, and more information on whether participants live in areas with high levels of air pollution exposure.

## Supplementary Information

**Additional file 1:** **Table 1.** Unadjusted Regression Analysis of IAP and OAP.

## Data Availability

The data (HINTS 4, Cycle 2) for the current study are available on the Health Information National Trends Survey website [https://hints.cancer.gov/data/survey-instruments.aspx#H4C2].

## Abbreviations

PM: Particulate Matter
US: United States
LMIC: Low-to-Middle-Income Countries
IAP: Indoor Air Pollution
OAP: Outdoor Air Pollution
HINTS: Health Information National Trends Survey
OR: Odds Ratio
CI: Confidence Intervals
