# Supplementary material for: Perception of worry of harm from air pollution: results from the Health Information National Trends Survey (HINTS)
Source: BMC Public Health. 2022 Jun 25;22:1254. doi: 10.1186/s12889-022-13450-z (PMC9233822; doi:10.1186/s12889-022-13450-z)
Supplement: Supplementary file 1 — Additional file 1: Table 1. Unadjusted Regression Analysis of IAP and OAP. [file 12889_2022_13450_MOESM1_ESM.docx]

**Table 1:** Unadjusted Regression Analysis of IAP and OAP

|  | **Indoor Air Pollution** | | | | | **Outdoor Air Pollution** | | | |
| --- | --- | --- | --- | --- | --- | --- | --- | --- | --- |
|  | **Some or a little** | | **A lot** | | | **Some or a little** | | **A lot** | |
|  | **vs.** | | **vs.** | | | **vs.** | | **vs.** | |
|  | **Not at all** | | **Not at all** | | | **Not at all** | | **Not at all** | |
| **Characteristic** | **OR** | **95% CI** | **OR** | | **95% CI** | **OR** | **95% CI** | **OR** | **95% CI** |
| **Sex** |  |  |  | |  |  |  |  |  |
| Male | 1.00 | - | 1.00 | | - | 1.00 | - | 1.00 | - |
| Female | 1.20 | 0.96 - 1.50 | 1.51 | | 1.02 - 2.24 | 1.38 | 1.03 – 1.86 | 1.83 | 1.23 – 2.71 |
| **Race/Ethnicity** |  |  |  | |  |  |  |  |  |
| Non-Hispanic White | 1.00 | - | 1.00 | | - | 1.00 | - | 1.00 | - |
| Hispanic | 1.30 | 0.88 - 1.93 | 3.05 | | 1.72 - 5.41 | 1.30 | 0.88 – 1.93 | 3.05 | 1.72 – 5.41 |
| Non-Hispanic Black | 1.05 | 0.76 - 1.45 | 1.74 | | 1.08 - 2.83 | 1.05 | 0.76 – 1.45 | 1.74 | 1.08 – 2.83 |
| Asian, Pacific Islander, and Other | 2.07 | 1.23 - 3.47 | 4.72 | | 1.94 – 11.47 | 2.07 | 1.23 – 3.47 | 4.72 | 1.94 – 11.47 |
| **Born in the USA** |  |  |  | |  |  |  |  |  |
| Yes | 1.00 | - | 1.00 | | - | 1.00 | - | 1.00 | - |
| No | 2.16 | 1.49 - 3.14 | 7.05 | | 4.67 - 10.63 | 2.13 | 1.43 - 3.18 | 7.32 | 4.96 - 10.80 |
| **Education** |  |  |  | |  |  |  |  |  |
| College Graduate or Post- Graduate Degree | 1.00 | - | 1.00 | | - | 1.00 | - | 1.00 | - |
| High School Diploma or Less | 1.21 | 0.92 - 1.58 | 2.18 | | 1.30 - 3.67 | 0.70 | 0.49 – 0.98 | 1.43 | 0.89 – 2.30 |
| Some College or Post-High School Vocational Training | 1.25 | 0.93 - 1.68 | 1.55 | | 0.93 - 2.59 | 1.01 | 0.76 - 1.35 | 1.02 | 0.67 - 1.56 |
| **Non-Metropolitan-Metropolitan Residence** |  |  |  | |  |  |  |  |  |
| Non-Metropolitan | 1.00 | - | 1.00 | | - | 1.00 | - | 1.00 | - |
| Metropolitan | 1.59 | 1.21 - 2.08 | 1.56 | | 0.95 - 2.58 | 1.61 | 1.17 - 2.21 | 1.98 | 1.22 - 3.24 |
| **Previous Family Cancer History** |  |  |  | |  |  |  |  |  |
| No | 1.00 | - | 1.00 | | - | 1.00 | - | 1.00 | - |
| Not Sure | 0.82 | 0.47 - 1.41 | 0.60 | | 0.27 - 1.36 | 0.94 | 0.53 – 1.65 | 0.75 | 0.35 – 1.61 |
| Yes | 1.01 | 0.76 - 1.35 | 0.77 | | 0.49 - 1.21 | 1.52 | 1.15 – 1.99 | 1.08 | 0.69 - 1.68 |
| **Previous lung disease diagnosis*** | |  |  | |  |  |  |  |  |
| Not Diagnosed with Lung Disease | 1.00 | - | 1.00 | | - | 1.00 | - | 1.00 | - |
| Diagnosed with Lung Disease | 1.32 | 0.84 - 2.09 | 1.81 | | 0.92 - 3.58 | 0.98 | 0.61 - 1.56 | 1.96 | 1.14 - 3.37 |
| **Likelihood of Getting Cancer** |  |  |  |  | |  |  |  |  |
| Neither Unlikely nor Likely | 1.00 | - | 1.00 | - | | 1.00 | - | 1.00 | - |
| Very Unlikely or Unlikely | 0.59 | 0.45 – 0.79 | 1.30 | 0.78 – 2.16 | | 0.67 | 0.45 – 1.00 | 1.30 | 0.75 – 2.26 |
| Very Likely or Likely | 1.07 | 0.77 – 1.50 | 1.75 | 1.13 – 2.73 | | 1.37 | 1.03 – 1.84 | 2.23 | 1.42 – 3.48 |
| *This includes chronic lung disease, asthma, emphysema, and/or chronic bronchitis | | | | | | | | | |
